# Enhanced Production by Terra-Sorb^®^ Symbiotic Biostimulant in Two Model Species Under Nitrogen Stress

**DOI:** 10.3390/plants14071087

**Published:** 2025-04-01

**Authors:** Laia Utgés-Minguell, Nuria Sierras-Serra, Cándido Marín, Marta Pintó-Marijuan

**Affiliations:** 1 Department of Evolutionary Biology, Ecology and Environmental Sciences, University of Barcelona, 08028 Barcelona, Spain; laiautgesminguell@gmail.com; 2 Plant Health R&D, Bioiberica, S.A.U, 08389 Palafolls, Spain; nsierras@bioiberica.com (N.S.-S.); cmarin@bioiberica.com (C.M.); 3 Institute of Research in Biodiversity (IRBio), University of Barcelona, 08028 Barcelona, Spain

**Keywords:** *Capsicum annuum*, horticultural production, *Lactuca sativa*, microbial biostimulant, nitrogen stress, photosynthetic performance, plant growth-promoting bacteria

## Abstract

The increasing soil pollution has accelerated the implementation of new agricultural regulations that significantly limit the use of synthetic nitrogen (N) fertilizers. Consequently, plants are likely to experience nutrient stress, leading to decreased productivity and potential threats to food security. To address these critical challenges, microbial-based biostimulant (BS) products, which utilize metabolites from microorganisms, offer a sustainable and eco-friendly solution to mitigate plant nutrient stress. This study evaluated the effects of the radicular application of a microbial-based BS containing L-α-amino acids on lettuce and pepper crops under two nitrogen regimes: optimal N availability and N stress (NS). Various parameters, including growth, production, soluble proteins, photosynthetic pigment content, and oxidative stress markers, were assessed. Under optimal N conditions, BS application enhanced commercial biomass in lettuce and vegetative biomass in pepper, indicating that BSs can reduce the need for nitrate uptake and endogenous amino acid synthesis, thereby conserving energy for other physiological processes. Despite BS application, NS conditions significantly reduced vegetative and reproductive growth in both species. However, BS treatment in pepper plants increased chloroplast pigments, improving light absorption and photosynthetic efficiency. The reduction in the carotenoid/chlorophyll ratio suggests efficient N allocation to growth and production. Thus, BS application proved effective in mitigating NS in pepper plants, enhancing pepper production, while under optimal conditions, it improved lettuce yield, particularly commercial biomass. These findings underscore the potential of symbiotic microbial-based BSs as a promising tool for sustainable agriculture under reduced N availability.

## 1. Introduction

In the coming decades, FAO studies forecast an increase in the global population exceeding 9.2 billion, requiring a 70% increase in food production to meet future demands [1]. Food security, a key focus of the Sustainable Development Goals, is becoming increasingly challenging due to the escalating impacts of climate change on agriculture. Modern agriculture has heavily relied on synthetic inputs, including pesticides and chemical fertilizers to maintain food security [2]. While these inputs have been crucial in stabilizing crop yields, the overuse of synthetic nitrogen fertilizers has raised relevant environmental concerns, including water contamination, soil degradation, and increased greenhouse gas emissions [3]. In response, the European Commission launched, in 2019, the European Green Deal which sets out policy initiatives to make Europe the first climate-neutral continent by 2050, including the Farm to Fork strategy, which aims to reduce the environmental impact of agriculture by decreasing chemical pesticide use and excessive synthetic nutrient inputs [4].

Nitrogen fertilization is crucial for maintaining global food production as it significantly enhances crop yields and quality. Nitrogen (N) is a plant macronutrient required for synthesizing proteins, nucleic acids, and chlorophylls, which are essential for growth and development [5]. Nitrogen deficiency stress (NS) impairs photosynthesis processes by reducing the biosynthesis of the photosynthetic enzymes involved in the Calvin–Benson Cycle, promotes stomatal limitation, and reduces light-harvesting protein availability as well as other proteins related to bioenergetics pathways such as Cytb6f or ATP synthase [6]. Therefore, the lack of nitrogen leads to lower energy production and reduced biomass, and crops fail to reach their full potential, resulting in lower yield and crop quality. On a global scale, this can threaten food security, which is exacerbated by climate change, and is expected to intensify hunger and malnutrition [7]. As a result, there is a growing need for sustainable practices in agriculture by adopting eco-friendly alternatives that optimize yields without compromising soil health or environmental sustainability [8]. Improved management practices that reduce dependency on synthetic inputs protect our natural resources while ensuring optimal productivity [9] such as biostimulants.

Biostimulants (BSs) are defined by the European Biostimulant Industry Council (EBIC) as substances or microorganisms that enhance plant nutrition efficiency, abiotic stress tolerance, and crop quality traits regardless of nutrient content. Due to their composition, they have been categorized and classified based on the substances and microorganisms that characterize them [10,11]. Among the various categories of biostimulants, protein hydrolysates (PHs), composed of amino acids and peptide mixtures, can be obtained from animal or plant by-products through chemical, enzymatic, or thermal hydrolysis, or a combination of these methods [11]. Enzymatic hydrolysis is considered a superior method for producing PHs because it preserves the biologically active form of amino acids which is the L-form, which is essential for crop growth and development [12]. This method of obtention avoids the conversion of the L-form to their less effective or even toxic D-forms, a drawback associated with chemical hydrolysis [13]. Additionally, the enzymatic mode of obtention is more sustainable and environmentally friendly compared to chemical methods, which can leave harmful residues such as sodium and sulfates [14]. PHs exhibit beneficial features in various physiological processes such as photosynthetic activity, nutrient assimilation, and translocation and positively impact quality parameters, enhancing fruit set and size in numerous crops [15,16]. Moreover, PHs contain bioactive peptides that can elicit hormone-like activities, influencing shoot and root growth and architecture, and contributing to the activation of both primary and secondary metabolism [17,18].

Another relevant BS group is the Plant Growth-Promoting Bacteria (PGPB), particularly the *Bacillus* genus, which promotes plant growth through nitrogen fixation [19] among other features and modes of action. They are also resistant to environmental stress conditions and are omnipresent in various types of soils [20,21]. *Bacillus* spp. can produce phytohormones such as abscisic acid, auxins, cytokinins, ethylene, jasmonic acid, and gibberellins [22] which are closely associated with the regulation of plant growth. Furthermore, these metabolites promote the induction of stress-related gene expression to maintain membrane stability and prevent the accumulation of reactive oxygen species (ROS) [19]. Another trait of *Bacillus* spp. is the facilitation of nutrient availability in the rhizosphere reducing the need for synthetic chemical fertilizers. Moreover, some studies have demonstrated increased uptake of N, P, and K when plants are under drought and salinity stress, showing that treatment with *Bacillus* increases the bioavailability of macronutrients [23,24]. One notable member of this genus is *B. velezensis,* which possesses strain-specific clusters of genes related to metabolites’ biosynthesis, which play significant roles in both pathogen suppression and plant growth promotion [21]. *Bacillus velezensis* PH-023 CECT 30677 is a bacterial strain that, with its metabolites, is formulated combined with L-α-amino acids from enzymatic hydrolysis in a commercially available microbial biostimulant named Terra-Sorb^®^ Symbiotic, (Bioiberica, S.A.U).

The objective of this study was to evaluate the effects of Terra-Sorb® Symbiotic on production and at a physiological level under N stress in two model horticultural species of agricultural economic interest: (1) a leafy species represented by lettuce (*Lactuca sativa* L.) and (2) a fruit producing species represented by pepper (*Capsicum annuum* L.). Moreover, we aimed to assess the potential of the stress mitigation by the PGPB in the microbial biostimulant based on L-α-amino acids application, as these crops require very high levels of fertilization to ensure adequate production. Therefore, this study considers the indirect provision of metabolites, L-α-amino acids, and symbiotic bacteria capable of interacting with the plant, with the hypothesis that the microbial BS will be able to compensate the provoked nitrogen stress with a more environmentally sustainable solution.

## 2. Results

To assess the effects of the nitrogen stress and the possible mitigation of the stress symptoms by BS application both, fruit horticultural species and leafy vegetables were included in the assay.

### 2.1. Nitrogen Stress Drastically Reduced Photosynthetic Activity, Pepper Production, and Plant Vegetative Biomass, but BS Application Promoted a Recovery of Both Vegetative and Reproductive Structures in Pepper Plants

**Figure 1:**
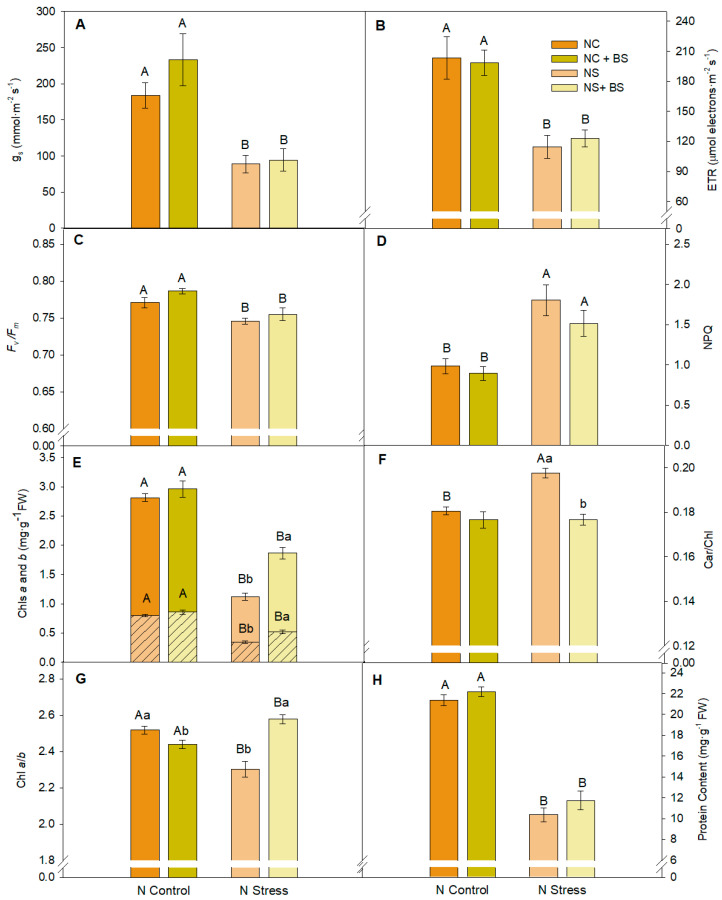
Effect of BS in photosynthetic performance under NC and NS treatments in pepper plants: stomatal conductance (g_s_; (**A**)), electron transport rate (ETR; (**B**)), PSII maximum efficiency (*F_v_*/*F_m_*; (**C**)), non-photochemical quenching (NPQ; (**D**)), total chlorophyll content (Chl *b* (lower bars) Chl *a* (upper bars); (**E**)), carotenoids/chlorophylls (Car/Chl; (**F**)), chlorophyll *a*/*b* ratio (Chl *a*/*b*; (**G**)), and protein content (**H**). Data show the mean ± SE (n = 8). Different letters indicate significant differences between nitrogen treatments (capital letters) or between BS applications (lower-case letters) with a P_value_ < 0.05.

The photosynthetic performance of pepper plants under NS conditions was affected by the N availability present in the substrate showing closed stomata (lower g_s_), slower electron transport rate (ETR), and also lower maximum PSII efficiency (*F_v_*/*F_m_*) (Figure 1A–C). Accordingly, N stress symptoms were also observed by higher NPQ and Car/Chl (Figure 1D,F), although symbiotic BS application played a significant role in reducing the Car/Chl in the NS plants to the NC values. Another stress symptom was the lower content of Chls, both Chl*a* and Chl*b* (Figure 1E), and total soluble proteins (Figure 1H) compared to control plants. The non-stressed plants showed no effect of the BS application in any of the previously described parameters. In fact, the Chl*a*/*b* ratio (Figure 1G) was the only parameter particularly affected by the BS application in the NC plants (decreased a 3.12% after BS application). However, the BS application significantly enhanced chlorophyll biosynthesis in the plants under NS from the 3rd week (Table S2, Figure S2). Specifically, chlorophyll *a* content experienced a significant rise in comparison with the non-treated plants, higher than 70% (Figure 1E), and chlorophyll *b* content increased by more than 50%, and consequently, the Chl*a*/*b* ratio increased a 12.04% after the BS application in the NS plants. Therefore, pepper plant vegetative functions were significantly reduced by the N stress, but the BS application allowed chlorophyll biosynthesis and Car/Chl ratio recovery, alleviating stress symptoms, and might have positive consequences on pepper fruit production.

**Figure 2:**
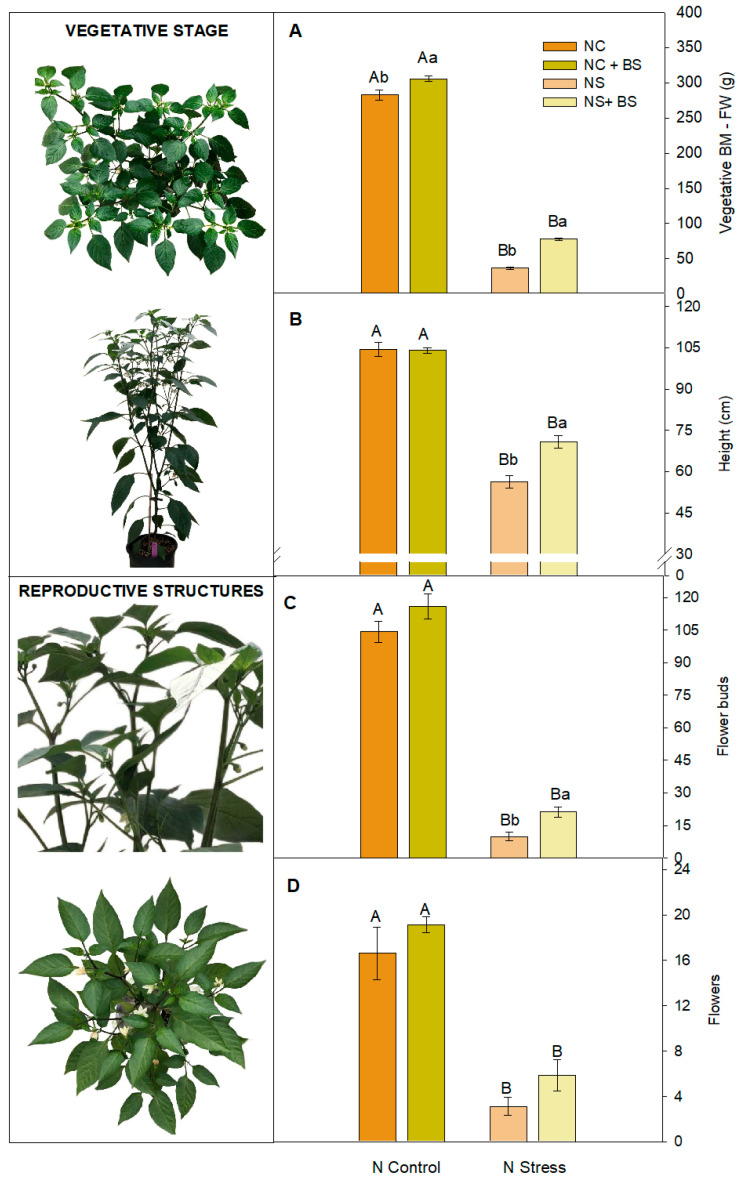
Vegetative and reproductive effects of BS under NC and NS treatments in pepper plants. Vegetative stage: vegetative BM-FW (**A**) and height (**B**); reproductive structures: flower buds (**C**) and flowers (**D**). Data show the mean ± SE (n = 8). Different letters indicate significant differences between nitrogen treatments (capital letters) or between BS applications (lower-case letters) with a P_value_ < 0.05.

The pepper plants were monitored weekly by quantifying multiple growth parameters (both vegetative and reproductive). Along the growing period, nitrogen stress provoked a strong shrinking on both the vegetative (Figures S3 and S4) and reproductive (Figures S5 and S6) growth parameters but the BS application had a significant mitigation of the stress symptoms from the 5th or 6th week (Tables S3–S5). Moreover, BS application also increased vegetative biomass in both nitrogen availabilities (Figure 2A). Nevertheless, the highest increase was observed in the N-stressed plants when BS was applied, showing a 110.5% vegetative biomass increase and around a 25.9% increase in pepper plant height (Figure 2B).

**Figure 3:**
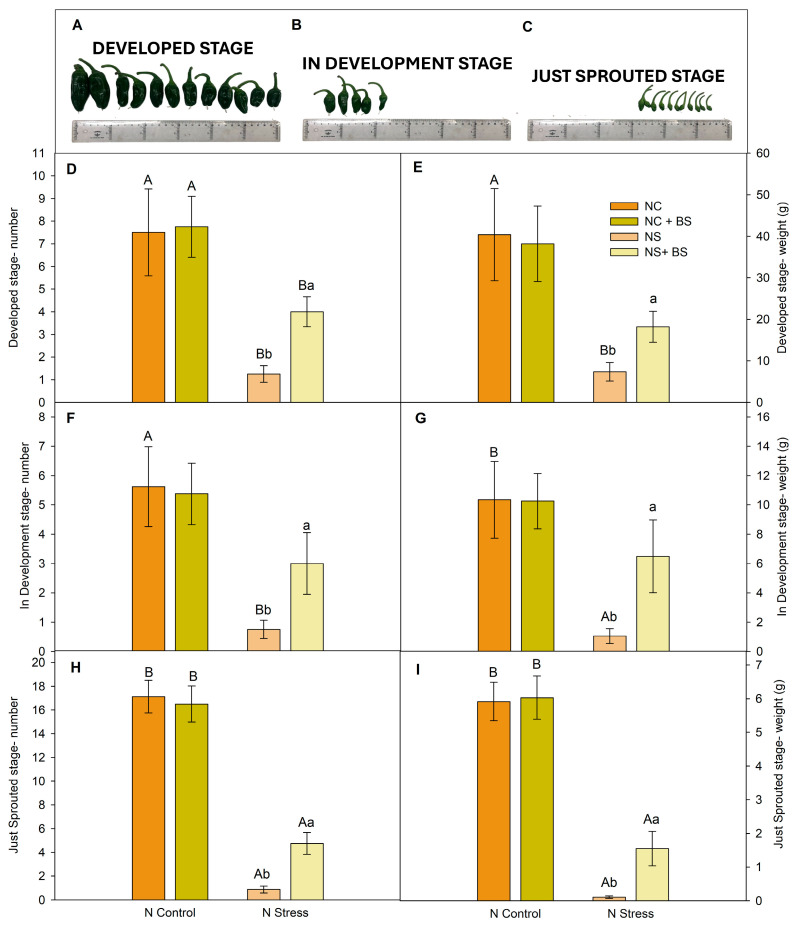
Images of the development stage in pepper plants (**A**–**C**) and the effect of BS under NC and NS treatments classified on the fruit set stage. Number of peppers: developed (**D**), in development (**F**), and just sprouted (**H**). Weight of peppers: developed (**E**), in development (**G**), and just sprouted (**I**). Data show the mean ± SE (n = 8). Different letters indicate significant differences between nitrogen treatments (capital letters) or between BS applications (lower-case letters) with a P_value_ < 0.05.

Regarding the reproductive parameters, BS application effects were observed palliating the N stress consequences and enhancing the number of flower buds since the 6th week (Table S5 and Figure 2C), fruit set, and fruit yield at any of the development stages (Figure 3). Furthermore, the results show a tendency to develop more flowers (Figure 2D), being significant in the 10th week (Figure S4B). The stress palliation in terms of fruit pepper production rise a 300% after BS application (Figure 3D) in the NS plants. When optimal N concentration (NC) was available, only a trend to form more flowers and flower buds was observed (Figure 2C,D and Figure S5), but was not reflected in higher production (Figure 3). Regarding pepper harvest at three development stages (Figure 3), very similar results were shown: (1) NS significantly decreased both the number and weight in the three development stages, and (2) the BS application enhanced the number and weight of the three development stages under NS. In fact, from the 10th week, the ‘just sprouted’ number of peppers (Table S6) was significantly more abundant in the BS-treated plants. Surprisingly, though the number of ‘just sprouted’ and ‘developed’ fruits of the NS + BS-treated plants was lower than NC, the BS application allowed no differences between NC + BS and NS + BS in the number and the weight of the ‘in development’ peppers (Figure 3F,G), as well as in the weight of the ‘developed stage’ peppers (Figure 3E). Therefore, the BS treatment significantly enhanced pepper fruit production in the NS plants.

### 2.2. In Lettuce Plants, BS Application Promoted Lower Need for Photoprotection (In NC), Lower Need for Protein Content (In NS), and Significantly Enhanced Commercial Production in Lettuce Plants

**Figure 4:**
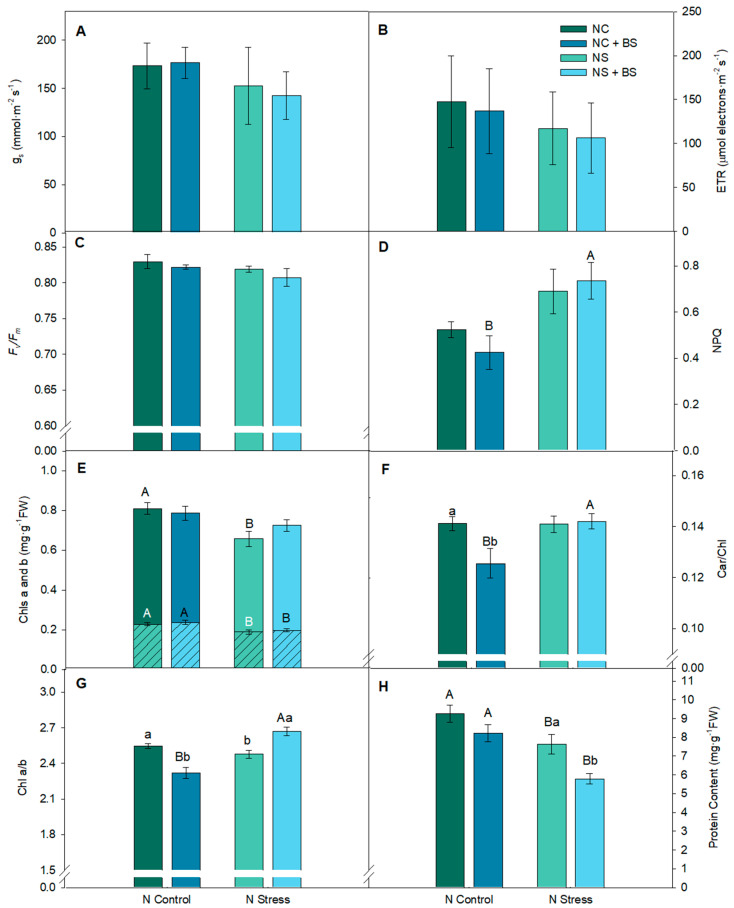
Effect of BS in photosynthetic performance under NC and NS treatments in lettuce: stomatal conductance (g_s_; (**A**)), electron transport rate (ETR; (**B**)), PSII maximum efficiency (*F_v_*/*F_m_*; (**C**)), non-photochemical quenching (NPQ; (**D**)), total chlorophyll content (Chl *b* (lower bars) Chl *a* (upper bars); (**E**)), carotenoids/chlorophylls (Car/Chl; (**F**)), chlorophyll *a*/*b* ratio (Chl *a*/*b*; (**G**)), and protein content (**H**). Data show the mean ± SE (n = 8). Different letters indicate significant differences between nitrogen treatments (capital letters) or between BS applications (lower-case letters) with a P_value_ < 0.05.

Most of the photosynthesis-related parameters assessed in the study (g_s_, ETR, and *F_v_*/*F_m_*) displayed no significant differences between the NC and NS treatments (Figure 4A–C) in lettuce plants. However, NS clearly affected energy dissipation by carotenoids in the PSII light-harvesting complex (LHC). For instance, NPQ and Car/Chl ratio (Figure 4D,E) were higher under NS (being significant differences between NC and NS in the BS-treated plants), indicating the need for enhanced photoprotective mechanisms under NS conditions. Another stress symptom was the significantly lower content of nitrogen-containing molecules in the NS plants compared to the NC plants: (1) chlorophylls, both Chl*a* and Chl*b* (Figure 4E), and (2) total soluble proteins (Figure 4H). Therefore, NS was affecting the lettuce plants’ performance moderately.

Regarding the BS application, the results showed minimal impact on photosynthetic parameters under both the NC and NS conditions. However, a significant 11% reduction in the Car/Chl ratio was observed in the BS-treated lettuce plants under NC (NC + BS) compared to the untreated plants (NC) (Figure 4F), suggesting that BS may reduce the need for carotenoids under optimal nitrogen conditions. Moreover, the BS application under NS enhanced chlorophylls’ biosynthesis (especially Chl*a*) that managed to have the same amount of Chl*a* as under control conditions (NC), promoting a significant rise in chlorophyll *a*/*b* ratio compared to the untreated plants (Figure 4F,G). SPAD weekly assessments demonstrated significant differences between both optimal N treatments (NC and NC + BS) compared to the N stress treatments (NS and NS + BS) from the 3rd week after transplant (Figure S7 and Table S7). Interestingly, protein content in both N availabilities experienced a decrease after BS application, being significant in the NS conditions with a 24.2% lower content in the NS + BS plants.

**Figure 5:**
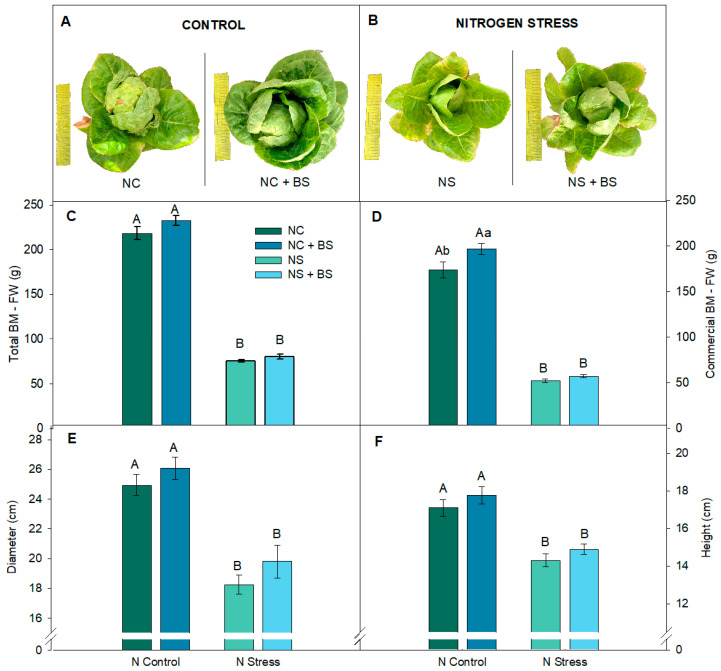
Images of the BS effects on harvested lettuce plants under NC (**A**) and NS (**B**) treatments and growth parameters. Production: total BM–FW (**C**), commercial BM–FW (**D**). Growth parameters: diameter (**E**), height (**F**). Data show the mean ± SE (n = 8). Different letters indicate significant differences between nitrogen treatments (capital letters) or between BS applications (lower-case letters) with a P_value_ < 0.05.

Lettuce growth development traits in both N availabilities (Figure 5) discovered a strong limitation in growth as a consequence of lower N conditions and NS significantly limited biomass, diameter, and height. For instance, height was significantly higher in NC than in NS since the 4th week in both BS treatments (Figure S8 and Table S8). BS application in the lettuces growing under NS did not lead to significant differences, although slight positive trends were observed in diameter and height. Under NC conditions, the BS-treated plants showed notable positive trends across all the measured parameters, including total BM, diameter, and height (Figure 5A,C,D and Figure S8), suggesting enhanced growth when BS was applied under optimal nitrogen availability. Moreover, under NC, commercial biomass (Figure 5B) was significantly higher after BS application, further boosting biomass compared to untreated plants.

## 3. Discussion

Currently, the agricultural sector is grappling with several challenges: (1) ensuring food availability for a growing global population, (2) enhancing resource use efficiency, and (3) minimizing the ecological impact of crop production [25]. To avoid the damaging consequences brought by the abusive use of N synthetic fertilizers supplied to increase the crop yield, plant BSs are considered promising and eco-friendly tools to reduce the use of synthetic fertilizers by improving nutrient use efficiency and uptake from the soil [26,27], but the new policies implementation bring plant nutrient stress consequences. In this study, two horticultural (lettuce and pepper) species were tested under severe N reduction as recommended for future crop management [28] complemented with the use of a microbial BS, based on L-α-amino acids and containing a *B. velezensis* bacterial strain with its fermentation metabolites.

Nitrogen (N) plays a main role in plant nutrition as it is involved in most physiological reactions and is a well-known key element present in several important biomolecules such as proteins, nucleic acids, hormones, or chlorophylls [29,30]. Regarding the photosynthetic performance in the lettuce plants, neither stomatal conductance nor photo-oxidative stress markers ETR, *F_v_*/*F_m_*, and NPQ were affected by low N availability, suggesting that there were no stress-induced perturbations in the photosynthetic apparatus and plants were not suffering physiological stress under varying N regimes. Navarro-León et al. [31]) also found no significant differences in parameters such as analyzed chlorophyll *a* fluorescence in lettuce under the same N doses in lettuce. However, in pepper plants, N deficiency provoked significant stress with a marked stomatal closure and severely affected photosystem photochemical efficiency. Lower ETR and *F_v_*/*F_m_* with higher NPQ and Car/Chl content were clear stress markers of N availability stress suffered by the pepper plants. ETR quantifies the electron transport rate (depending on the *Φ_PSII_* and the light incidence) that is used in photochemistry and *F_v_*/*F_m_* reflects the potential maximum PSII efficiency, being a sensitive indicator of plant photosynthetic performance, with optimal values typically ranging from 0.79 to 0.83 [32,33]. Our study recorded an average of 0.81 across all treatments in lettuce plants but N-stressed pepper plants without BS application presented values under 0.75. Several abiotic stresses require antioxidant protection mechanisms to safeguard the photosynthetic mechanisms such as thermal dissipation via NPQ is necessary to avoid photo-oxidation and protect PSII by preventing reactive oxygen species (ROS) formation [34]. Carotenoids play a crucial role in photoprotection, both in thermal dissipation (especially zeaxanthin and lutein) and in direct ROS scavenging [34]. In our study, N stress provoked, in the two different species, a reduction in the composition of the analyzed photosynthetic pigments: chlorophylls and carotenoids. The induction of stomatal closure and reduction in total chlorophyll and carotenoid content was also observed by Huang et al. [35] when N was limited in rice plants and by [25] in greenhouse-grown basil plants.

The BS application only reduced photoprotection needs by decreasing the Car/Chl ratio under NC conditions in lettuce plants and under NS in pepper plants, although the Chl*a* and Chl*b* content were also enhanced. Some studies proved that the application of amino acid-based BS boosted chlorophyll and carotenoid content in several species under nutrient reductions (Table S9) [36,37]. Our observations evidenced that the application of microbial BSs can manage lower N availability at the root level maintaining the biosynthesis of chlorophylls *a* and *b*. The presence of exogenous protein hydrolysates and amino acids such as glutamate, precursors of chlorophyll biosynthesis, restored chlorophyll levels to perform optimal photosynthesis rates under N deficiency [31]. It has been studied in tomatoes under reduced NPK nutrition that foliar applications of polysaccharides, protein hydrolysates, and humic acids-BS can also preserve chlorophyll content at optimal levels [36].

In both model species, N availability reduction showed a significant reduction in the measured N-containing molecules: chlorophylls and soluble proteins independently of the BS treatment. With low N available in soil, once N is assimilated into the plant and reduced to NH_4_^+^, lower availability of NH_4_^+^ will restrict the biosynthesis of amino acids and future soluble proteins. Hence, the observed reduction in biomass can be suggested by a limited protein biosynthesis attributed to a decrease in N availability in the substrate. As has been demonstrated by Gao et al., [38] protein content shows a direct relationship with biomass production.

BS treatment to lettuce plants showed an unexpected decrease in soluble protein content, being significant under nitrogen stress. This observation strongly contrasts with the findings from other studies. For instance, Irani et al. [39] investigated the application of several BSs (amino acids, humic acids, fulvic acids, and seaweed extracts) under drought stress conditions in grapevine and showed that BS application significantly increased soluble protein content. Furthermore, Carillo et al. [17] confirmed that in spinach, under different N availabilities, the foliar application of protein hydrolysate-based BS promoted an increase in protein content. Therefore, our findings are not in accordance with these publications and the disruptive results obtained in our study must be understood.

Protein biosynthesis needs N to build the amino acid building blocks, whose biosynthesis uses energy (adenosine triphosphate, ATP) and reducing power (NADPH/NADH) both to reduce NO_3_ from the soil to NH_4_^+^ and to synthesize the wide range of necessary enzymes involved in such reduction [40]. The required energy to assimilate N from the soil is provided by the glycolysis of sucrose at both roots and leaves. The triose phosphate resulting from the Calvin Cycle is the precursor of sucrose or other carbohydrates that might subsequently be converted into starch to store potential cell energy. RubisCO plays a crucial role in the carboxylation reaction to assimilate CO_2_ molecules in the Calvin Cycle; this protein represents around 40% of total soluble proteins in most leaf plants. The following reduction phase of the Calvin Cycle expends three molecules of NADPH and two molecules of ATP, finally allowing the synthesis of the triose phosphate molecules. Briefly, the creation of amino acids and proteins is an expensive biosynthetic pathway that needs a huge amount of ATP, carbohydrates, and several enzymes. However, BS-treated plants may bypass these metabolic processes, as amino acids are already available for the plant with an income of the BS product. The available L-α-amino acids can be translocated and used directly, reducing the need for RubisCO and other soluble proteins involved in these physiological processes which provokes a plant’s energy saving that can be used in other physiological processes. In our study, BS treatment in lettuce plants allowed the metabolic process to bypass the nitrogen stress, leading to lower protein content.

Analyzing the effects on growth, N stress decreased biomass in both the studied species as N soil availability is directly related to growth parameters and biomass production as is concluded in previous reports in cucumber [41], in tomato [42], and in canola [43]. Regarding BS application, on one side, the lettuce plants showed no effect on total biomass under any of the N availability conditions, indicating a non-limiting nitrogen concentration in this species not even under strong N reduction. However, commercial BM significantly increased due to BS application under NC. Nonetheless, BS application on pepper plants significantly increased the vegetative BM on both N regimes and fruit production under NS. This enhancement can be attributed to the exogenous L-α-amino acids pool provided by the BS which reduced the need for uptaking nitrate and the synthesis of endogenous amino acids and enzymes, conserving energy for other physiological processes. The higher amounts of early fruit set might be increased by the presence of metabolites from the microbial BS optimizing N use and allowing it to be spent to promote fruit development. Likewise, after spraying moringa leaf extract, seaweed extract, and fulvic acid BSs in apple trees, Mosa et al. [44] found improvement in fruit set and fruit yield due to better nutrient availability. Consequently, plants can use the available N more efficiently, promoting plant growth and increasing essential molecules such as chloroplast pigments. This increases the antenna pigments, allowing more light absorption by larger LHC used to enhance photosynthetic processes resulting in a final increase in pepper production. Therefore, the decrease in the Car/Chl ratio after the BS treatment showed that available N was used to increase vegetative growth and fruit production as LHC was fully equipped. Recently, several reviews have highlighted the combination of PGPB with non-PGPB biostimulant ingredients as a powerful tool to enhance growth and production under various abiotic stresses [45,46,47,48,49]. However, there are few studies focusing specifically on nutrient deficiencies (Table 1). Mainly, protein hydrolysates have been shown to increase yield and positively impact photosynthesis-related processes, including the electron transport chain and gas exchange. Additionally, some seaweed extracts or melatonin-based BS have promoted higher yields and contributed to reducing stress markers such as antioxidant enzymes. It is crucial to continue investigating the mechanisms of action for the available BSs on the market, as well as developing new products with the potential to overcome various stresses affecting crops globally.

In the current study, BS composition played a key role in promoting vegetative and reproductive growth due to the presence of metabolites generated by *B. velezensis* such as (1) gibberellins (GAs) which are involved in stem elongation [50]; (2) indole-3-acetamide (IAM), a precursor of indol-3-acetic acid (IAA) which is responsible for controlling several physiological processes such as cell elongation and division and play a pivotal role in plant development of shoots and roots [50,51]; and (3) pantothenic acid (vitamin B5) which promotes vegetative development, among others. When N was less available, the *B. velezensis* strain was performing mutualistic relations with the plant, enhancing nitrogen concentration in plant tissues by N_2_ fixation from the atmosphere [20]. Regarding reproductive growth in pepper plants, both fruit yield and fruit setting precocity were enhanced only under N reduction stress. Our results are in accordance with Mesa et al. [37], whose application of a BS based on enzymatically hydrolyzed animal protein combined with low N priming, resulted in a significant increase in fruit production, effectively doubling the yield. Finally, the combined effect of metabolites, such as IAM and GA_4_, acted as signaling molecules influencing various aspects of plant growth (in both root and shoot) allowing higher biomass rates [16,52].

**Table 1:** Effect of biostimulants under nutrient deficiency stress conditions.

Biostimulant		Crop	Application	Stress	Results	References
PHs	Amino acids, soluble sugars, and phenols	Jute	Foliar	Full Nutrient Stress	Increased yield, enhanced CO_2_ assimilation, chlorophyll content, and nitrogen distribution	[53]
		Basil	Root	Full Nutrient Stress	Enhanced yield and functional quality attributes	[54]
		Lettuce	Foliar	Nitrogen Stress	Improved yield, chlorophyll content, N-uptake efficiency, and HAA	[55]
		Spinach	Foliar	Nitrogen Stress	Improved yield, chlorophyll content, N-uptake efficiency, and LAA	[55]
	Amino acids	Tomato	Root	Nutrient Stress	Enhanced primary and lateral root growth, by stimulating the biosynthesis of salicylic acid under stress	[56]
		Tomato	Root	Nitrogen Stress	Tendency to improve total fruit production	[37]
	Free L-α-amino acids and organic matter	Lettuce	Root	Nitrogen Stress	Enhanced photosynthesis, total N, and increased biomass production	[31]
Seaweed extract		Lettuce	Foliar	Nitrogen Stress	Increased photochemical efficiency and marketable fresh yield	[57]
		Tomato	Foliar	Nutrient Stress (NPK)	Decreased SOD and POD; maintained fruit quality and yield	[36]
Melatonin		Soybean	Root	Nitrogen Stress	Increased biomass, stem diameter, total leaf area, root nodule number, SOD, GPX and CAT	[58]
		Cucumber	Foliar	Nitrogen Stress	Enhanced activity of enzymes involved in N metabolism and N assimilation efficiency	[59]
Others	Betaines, alginic acid, and kaidrin	Lettuce	Foliar	Nitrogen Stress	Increased yield	[60]

Abbreviations: catalase (CAT); guaiacol peroxidase (GPX); hydrophilic antioxidant activity (HAA); lipophilic antioxidant activity (LAA); peroxidase (POD); protein hydrolysates (PHs); superoxide dismutase (SOD).

## 4. Materials and Methods

### 4.1. Plant Material and Experimental Design

Two different species were grown in the greenhouse of the Servei de Camps Experimentals (University of Barcelona, Spain) from the 27 February to the 8 May 2024. The selected model plant species were (1) a horticultural leafy species represented by the Little Gem lettuce (*Lactuca sativa* L. var. Little Gem Cerler) and (2) a fruit-producing species represented by Padrón pepper (*Capsicum annuum* L. var. Longum). Seedlings from both species were transplanted at the 4–5 true-leaf stage into a substrate composed of peat, perlite, and vermiculite in a 2:1:1 ratio. Both species seedlings were obtained from Semilleros Deitana S.L. (Murcia, Spain). During the experiment, temperature and relative humidity inside the greenhouse were recorded (Figure S1).

The trial was designed to assess the application or absence of a PGPB biostimulant: Terra-Sorb^®^ Symbiotic (TSS), combined with two nitrogen regimes. The plants were irrigated with Hoagland Solution (HS) following the nutrient recipe developed by Hoagland and Arnon (1938) [61] adapted to our studied species and using different contents on nitrogen compounds (Table S1). The nitrogen contents in both nitrogen regimes were: (1) Nitrogen Control (NC) with 8 mmol N L^−1^; (2) nitrogen stress (NS) with 2.4 mmol N L^−1^. Moreover, half of each plant species, lettuces and peppers, received radicular Terra-Sorb^®^ Symbiotic biostimulant (+BS) application (as indicated in Figure S1). This design resulted in a total of four treatment groups per species (NC, NC + BS, NS, and NS + BS). To ensure statistical robustness, each treatment group was replicated in 8 different plants randomly distributed throughout the greenhouse (n = 8).

The strain PH-023 has been chosen among extremophile bacteria for its great growth capacity and development in difficult soil conditions for agriculture. PH-023 strain can fix atmospheric nitrogen, produce siderophores, synthesize biosurfactants, and promote the production of phytohormones, especially auxins. PH-023 is produced by fermentation technology using partially as a nitrogen source the same carrier, a hydrolyzed protein obtained through enzymatic hydrolysis, where the bacteria are formulated. The product contains amino acids, the bacteria PH-023, as well as the metabolites synthesized through the fermentation process. Specifically, Terra-Sorb^®^ Symbiotic contains 6% *w*/*w* of L-α-free amino acids, 10^7^ cfu/mL PH-023, and 8% *w*/*w* of organic matter with a density of 1.12 kg L^−1^ and a pH of 5.0. Finally, the aminogram of the product (in g kg^−1^) is as follows: Asp (4.3), Ser (3.2), Glu (5.9), Gly (5.8), His (1.7), Arg (3.2), Thr (3.6), Ala (3.9), Pro (2.8), Cis (0.2), Tyr (3.6), Val (4.0), Met (3.8), Lys (3.9), Ile (3.0), Leu (5.3), Phe (2.9), and Trp (0.5).

All the treatments were applied through root irrigation to the substrate at intervals of 10 to 12 days throughout the experimental period. Therefore, lettuce, with a growth duration of 45 days, received a total of 4 BS applications, while pepper, with a growth duration of 65 days, received 6 applications (Figure S1). The dose was adjusted based on the planting frame: lettuce plants received 0.13 mL BS/plant and pepper plants received 1 mL BS/plant. Therefore, as Terra-Sorb^®^ Symbiotic provides 2.5 mmol N L^−1^, the extra nitrogen supplied to each treatment due to BS application were as follows: (1) in lettuce plants 1.3 µmol N/plant; (2) in pepper plants 15 µmol N/plant.

### 4.2. Growth Quantification

Lettuce growth was quantified weekly (from 1st to 5th week) by number of mature leaves and height. Moreover, at harvest, diameter, total biomass (BM), and commercial biomass in fresh weight (FW) were assessed. The pepper plants were monitored weekly depending on their developmental stage. Along with the experiment, plant height was measured. During the first stage of plant development (from the 1st to 7th week), the number of mature (fully developed) and developing leaves was quantified. When the reproductive stage began (from the 4th week), the number of flower buds and open flowers was also counted, continuing until the end of the experiment. In the 8th week, fruit set began and the number of just sprouted peppers was counted and classified based on size, distinguishing between those larger and smaller than 1 cm. At the end of the trial, the total BM of the vegetative tissue of each plant was measured, and the harvested fruits were classified depending on their developmental stage: (1) ‘just sprouted’ peppers (length ≤ 1 cm); (2) ‘in development’ peppers (length 1 < x ≥ 3); (3) ‘developed’ peppers (length ≥ 3 cm and Ø ≥ 1.5 cm). To assess the pepper production across the four treatments, the weight for every group was measured, and all the fruits were counted.

### 4.3. Photosynthesis Related Parameters

At the end of the trial, a MINI-PAM-II modulated pulse fluorimeter combined with a porometer (Heinz Walz GmbH, Effeltrich, Germany) was used to measure stomatal conductance (g_s_) and chlorophyll fluorescence. Measurements under light conditions were used to quantify g_s_, the relative efficiency of PSII (*Φ_PSII_*), and to obtain the *F’_m_* for non-photochemical quenching (NPQ) calculation. Afterward, leaves were dark-adapted for more than 20 min, and measurements of maximum efficiency of PSII (*F_v_*/*F_m_*) were taken in the dark. Finally, NPQ was calculated as (*F_m_* − *F’_m_)*/*F’_m_* [62].

Photosynthetic pigments were measured at the end of the trial on fully developed lettuce and pepper leaves, which were immediately frozen into liquid nitrogen and stored at –80 °C until analysis. To determine the chlorophyll *a* (Chl*a*), chlorophyll *b* (Chl*b*), and total carotenoids (Car), 100 mg of lettuce frozen leaves and 50 mg of pepper frozen leaves were ground to powder. Extraction was performed by adding 1 mL of cold pure methanol. After the vortex, the extracts were ultrasonicated (Bransonic ultrasonic bath 2800, Emerson Industrial, Ferguson, MO, USA). The supernatant was collected, and the pellet was re-extracted once with the same procedure, pooling at the end both the supernatants to proceed with measurements. The absorbance of the diluted extracts (1:4 for lettuce and 1:5 for pepper) was measured at wavelengths of 750, 665.2, 652.4, and 470 nm using a UV-visible spectrophotometer (Cecil Aquarius CE7400, Cecil Instruments, Cambridge, UK) according to Lichtenthaler [63].

Moreover, total chlorophyll (Chl) content was estimated weekly with a SPAD-502 Plus chlorophyll meter (Konica Minolta Business Solutions Europe GmbH, Chicago, IL, USA). Measurements were made in three fully developed leaves per plant and the average was used as individual value.

### 4.4. Total Soluble Proteins

The total soluble protein content in fully developed leaves was determined with 100 mg of frozen lettuce leaves and 75 mg of frozen pepper leaves. Plant tissue was ground to powder, and the extraction of soluble proteins was performed using 1 mL of phosphate-buffered saline and vortexing. The extracts were centrifuged, and the supernatant was collected. The absorbance of 50 µL supernatant reacted with the Bradford solution was measured at 595 nm using the same UV-Vis spectrophotometer (UV/VIS3000, DINKO Instruments, Barcelona, Spain), and the protein content was determined using a standard curve performed with lyophilized bovine serum albumin (BSA).

### 4.5. Statistical Analysis

Program IMB SPSS Statistics 29 for Macintosh (SPSS Inc, Chicago, IL, USA) was used for statistical analysis. An analysis of the variance (ANOVA) was used to compare mean values and set differences between the nitrogen stress treatments and differences between the BS application treatments. The significance level was set at 95% (*p* < 0.05). Significantly different means were marked with different letters. The SigmaPlot 10.0 (Systat software, Palo Alto, CA, USA) software was used to create all the figures in this publication.

## 5. Conclusions

Worldwide food demand is increasing, while at the same time, high amounts of synthetic N fertilizers used in agriculture are polluting our ecosystems. Fertilizer reduction to avoid environmental drawbacks provokes plant stress with the consequent severe productivity drop. Thus, environment-friendly tools must be implemented in agriculture regulation policies. In this study, our results proved that microbial BS benefits help to maintain or even improve production. The two model horticultural species demonstrated that nitrogen stress reduced biomass and productivity, but different modes of action were used after BS application: (1) on one side, pepper plants growing under nitrogen stress, showed a significant increase in vegetative growth (BM and height) and reproductive parameters (production and fruit setting); (2) on the other side, lettuce commercial biomass was enhanced only when plants were under optimal N availability. Therefore, the plants treated with microbial BS showed higher commercial outputs, both commercial biomass in lettuce and enhanced production of all the different pepper development stages. As previously suggested, the metabolites of the microbial BS acted as growth promoters, enhancing plant development and the plant’s metabolic efficiency. In conclusion, symbiotic BS will provide a sustainable solution to face food production in the near future and counteract the stress caused by reduced fertilization, specifically the reduction in synthetic nitrogen sources due to current regulations and trends promoted by society.

## Data Availability

Data will be made available upon request.

## Supplementary Materials

The following supporting information can be downloaded at https://www.mdpi.com/article/10.3390/plants14071087/s1, Table S1: Macronutrient and micronutrient composition of fertigation solution; Figure S1: Temperature and relative humidity in the greenhouse during the experiment; Table S2 and Figure S2: Chlorophyll content of pepper plants assessed weekly; Table S3 and Figure S3: Height of pepper plants assessed weekly; Table S4 and Figure S4: Number of ‘in development leaves’ (A) and ‘mature leaves’ (B) of pepper plants assessed weekly; Table S5 and Figure S5: Number of flower buds (A) and developed flowers (B) of pepper plants assessed weekly; Table S6 and Figure S6: Number of just sprouted pepper fruits smaller or equal (A) to 1 cm or bigger (B) than 1 cm assessed weekly; Table S7 and Figure S7: Chlorophyll content of lettuce plants assessed weekly; Table S8 and Figure S8: Height (A) and number of Mature Leaves (B) of the lettuce plants assessed weekly.

## Abbreviations

The following abbreviations are used in this manuscript:

ATP	adenosine triphosphate
BM	total biomass
BS	biostimulants
Car	total carotenoids
Chl*a*	chlorophyll *a*
Chl*b*	chlorophyll *b*
ETR	electron transport rate
FW	fresh weight
*F_v_*/*F_m_*	maximum efficiency of PSII
g_s_	stomatal conductance
LHC	light-harvesting complex
NC	Nitrogen Control Treatment
NPQ	non-photochemical quenching
NS	Nitrogen Deficiency Stress Treatment
PGPB	Plant Growth-Promoting Bacteria
PHs	protein hydrolysates
ROS	reactive oxygen species
*Φ_PSII_*	relative efficiency of PSII
