# Supplementary material for: Enhanced Production by Terra-Sorb® Symbiotic Biostimulant in Two Model Species Under Nitrogen Stress
Source: Plants (Basel). 2025 Apr 1;14(7):1087. doi: 10.3390/plants14071087 (PMC11991472; doi:10.3390/plants14071087)
Supplement: Supplementary file 1 [file plants-14-01087-s001.zip › plants-3529016-supplementary.pdf]

SUPPLEMENTARY MATERIAL - PLANTS

Enhanced production by Terra-Sorb® Symbiotic biostimulant in two model species under nitrogen stress.

Laia Utgés-Minguell[1], Nuria Sierras-Serra[2], Cándido Marín[2] and Marta Pintó-Marijuan[1,3*]

[1] Department of Evolutionary Biology, Ecology and Environmental Sciences, University of Barcelona, 08028 Barcelona, Spain.

[2] Plant Health R&D, Bioiberica, S.A.U, 08389 Palafolls-Barcelona, Spain.

[3] Institute of Research in Biodiversity (IRBio), University of Barcelona, 08028 Barcelona, Spain.

Table S1. Macronutrient and micronutrient composition of fertigation solution considering both different nitrogen regimes, N-30% and N-100%, for lettuce and pepper species.

	LETTUCE		PEPPER	
Macronutrients	NS (mmol·L^{-1})	NC (mmol·L^{-1})	NS (mmol·L^{-1})	NC (mmol·L^{-1})
NO_3^-	2.1	7	2.1	7
NH_4^+	0.3	1	0.3	1
PO_4^{2-}	1	1	2	2
K^+	3	3	6	6
Ca^{2+}	2	2	4	4
Mg^{2+}	0.5	0.5	1	1

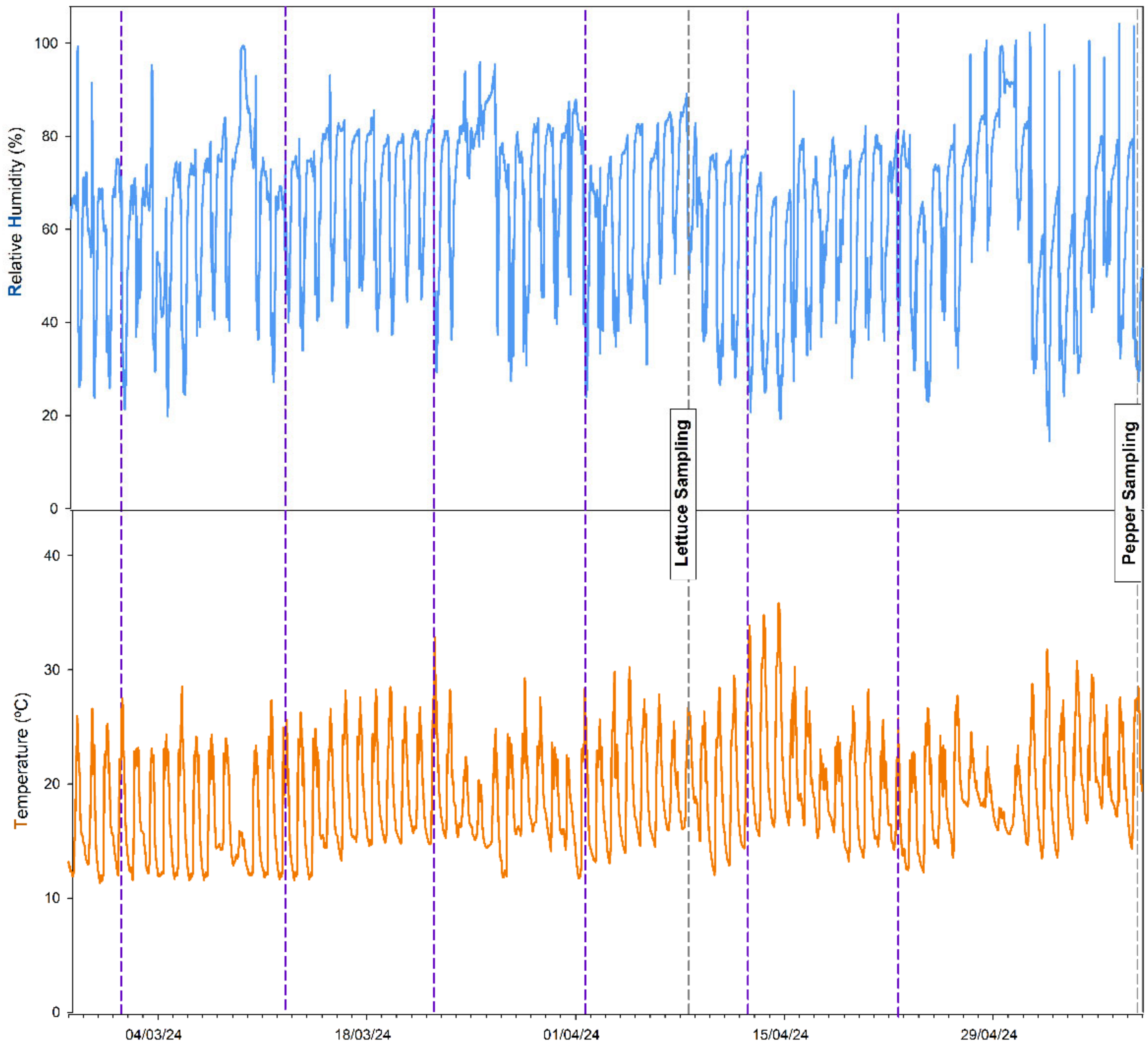

Figure S1. Temperature and Relative Humidity in the greenhouse during the experiment. Purple dashed lines represent the BS application, grey dashed lines represent the sampling days on both species.

Table S2. Chlorophyll content of pepper plants weekly assessed by SPAD spectroradiometer Index in the four treatments. NC: Nitrogen Control; NS: Nitrogen Stress; +BS: Biostimulant application. Data show the mean ± SE (n=8). Different letters indicate significant differences between nitrogen treatments (capital letters) or between BS applications (lower-case letters) with $P_{value} < 0,05$.

Week	NC	NC+BS	NS	NS+BS
1	33,24 ± 1,44	32,91 ± 1,00	32,03 ± 1,02	31,64 ± 0,96
2	37,72 ± 1,27	35,00 ± 0,68	35,03 ± 1,03	33,91 ± 0,86
3	43,13 ± 0,49 $^{A\ a}$	45,51 ± 0,43 $^{A\ b}$	36,94 ± 0,95 $^{B\ a}$	41,00 ± 0,87 $^{B\ b}$
4	45,40 ± 0,63 $^{A\ a}$	48,31 ± 1,04 $^{A\ b}$	37,71 ± 0,40 $^{B\ a}$	42,91 ± 0,59 $^{B\ b}$
5	48,26 ± 0,66 ^{A}	48,12 ± 1,29 ^{A}	40,20 ± 0,71 ^{B}	42,18 ± 0,74 ^{B}
6	47,62 ± 0,75 $^{A\ a}$	50,93 ± 0,88 $^{A\ b}$	38,76 ± 0,68 $^{B\ a}$	45,13 ± 0,83 $^{B\ b}$
7	47,48 ± 0,92 ^{A}	50,33 ± 1,03 ^{A}	38,55 ± 0,76 $^{B\ a}$	45,27 ± 0,81 $^{B\ b}$
8	51,34 ± 1,55 ^{A}	54,72 ± 1,10 ^{A}	40,05 ± 0,70 $^{B\ a}$	46,20 ± 0,73 $^{B\ b}$
9	54,38 ± 0,74 $^{A\ a}$	57,66 ± 0,98 $^{A\ b}$	40,92 ± 0,96 $^{B\ a}$	47,95 ± 1,37 $^{B\ b}$
10	56,98 ± 0,73 ^{A}	58,30 ± 1,35 ^{A}	41,46 ± 1,28 $^{B\ a}$	48,80 ± 0,74 $^{B\ b}$
11	59,40 ± 0,67 ^{A}	60,40 ± 0,79 ^{A}	43,40 ± 0,74 $^{B\ a}$	50,20 ± 1,05 $^{B\ b}$

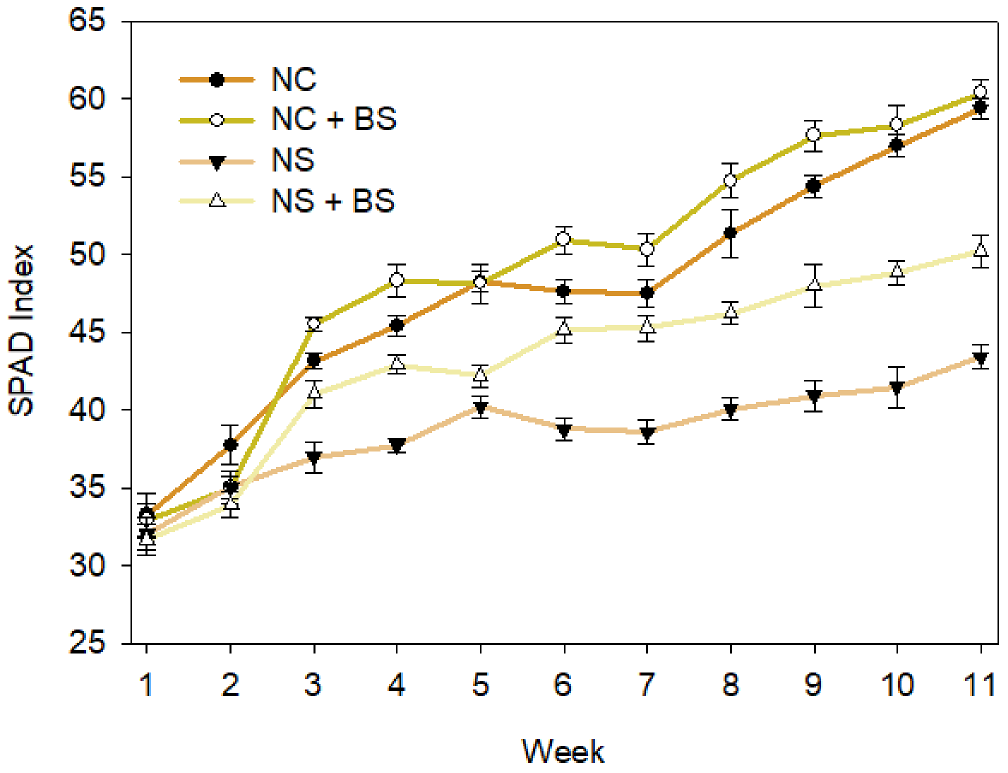

Figure S2. Chlorophyll content of pepper plants weekly assessed by SPAD spectroradiometer Index in the four treatments. NC: Nitrogen Control (circle); NS: Nitrogen Stress (triangle); +BS: Biostimulant applications (empty symbol). Data show the mean ± SE (n=8).

Table S3. Height of pepper plants weekly assessed in the four treatments. NC: Nitrogen Control; NS: Nitrogen Stress; +BS: Biostimulant application. Data show the mean ± SE (n=8). Different letters indicate significant differences between nitrogen treatments (capital letters) or between BS applications (lower-case letters) with $P_{value} < 0.05$.

Week	NC	NC+BS	NS	NS+BS
1	12.85 ± 0.26	13.06 ± 0.27	13.10 ± 0.23	12.86 ± 0.38
2	15.15 ± 0.25	15.01 ± 0.33	15.21 ± 0.21	14.98 ± 0.31
3	18.19 ± 0.38	17.89 ± 0.37	17.25 ± 0.25	18.10 ± 0.48
4	25.01 ± 0.64 A	24.11 ± 0.59	21.53 ± 0.38 B	22.73 ± 0.55
5	34.58 ± 2.15 A	35.31 ± 0.95 A	26.68 ± 0.55 B a	30.20 ± 1.01 B b
6	46.04 ± 1.44 A	43.06 ± 0.61 A	30.33 ± 0.84 B a	36.61 ± 1.17 B b
8	76.19 ± 2.09 A	73.94 ± 1.15 A	45.25 ± 0.92 B a	55.13 ± 1.27 B b
9	87.91 ± 2.31 A	85.75 ± 1.46 A	48.56 ± 1.09 B a	62.00 ± 1.74 B b
10	96.25 ± 2.97 A	96.44 ± 1.64 A	50.94 ± 1.31 B a	66.81 ± 2.27 B b
11	104.44 ± 2.47 A	104.00 ± 0.89 A	56.19 ± 2.38 B a	70.75 ± 2.18 B b

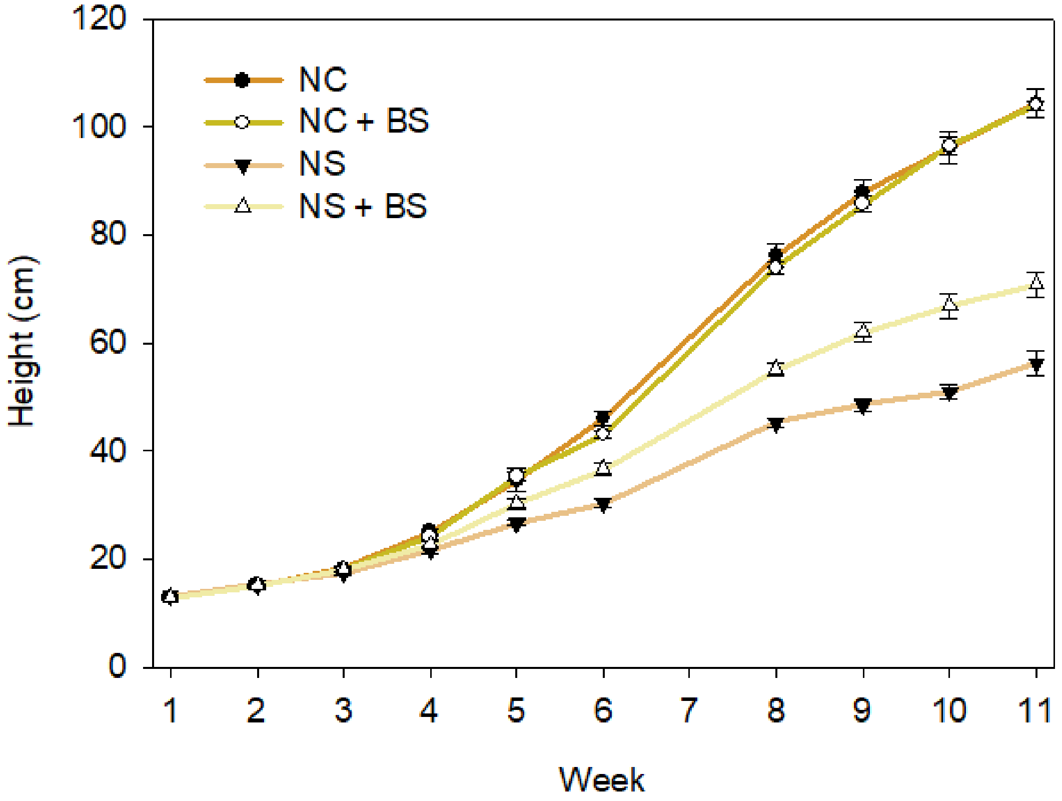

Figure S3. Height of pepper plants weekly assessed in the four treatments. NC: Nitrogen Control (circle); NS: Nitrogen Stress (triangle); +BS: Biostimulant applications (empty symbol). Data show the mean ± SE (n=8).

Table S4. Number of 'in development leaves' (A) and 'mature leaves' (B) of pepper plants weekly assessed during the plant development first stage (from 1st to 7th week) in the four treatments. NC: Nitrogen Control; NS: Nitrogen Stress; +BS: Biostimulant application. Data show the mean ± SE (n=8). Different letters indicate significant differences between nitrogen treatments (capital letters) or between BS applications (lower-case letters) with $P_{value} < 0.05$.

	Week	NC	NC+BS	NS	NS+BS
In Development Leaves	3	6.75 ± 1.24 A	6.50 ± 0.80 A	2.13 ± 0.13 B	2.38 ± 0.42 B
	4	10.50 ± 1.13 A	10.50 ± 0.53 A	4.63 ± 0.38 B	7.13 ± 1.13 B
	5	24.13 ± 1.34 A	25.88 ± 1.11 A	11.38 ± 1.36 B a	17.25 ± 1.89 B b
	6	37.25 ± 1.69 A	39.13 ± 1.80 A	14.00 ± 1.25 B a	22.63 ± 1.75 B b
	7	66.63 ± 1.76 A	68.50 ± 2.49 A	19.13 ± 1.52 B a	32.38 ± 2.66 B b
Mature Leaves	1	8.63 ± 0.46	8.25 ± 0.16	7.75 ± 0.16	8.13 ± 0.35
	2	11.88 ± 0.72	11.25 ± 0.16	10.50 ± 0.19	11.13 ± 0.58
	3	12.13 ± 0.74	11.00 ± 0.38	10.50 ± 0.33	10.88 ± 0.58
	4	14.63 ± 0.68 A	12.88 ± 0.55	11.88 ± 0.40 B	12.38 ± 0.53
	5	20.50 ± 2.06 A	17.50 ± 1.25	12.50 ± 0.27 B a	15.25 ± 1.13 b
	6	20.38 ± 1.27 A	22.38 ± 1.99 A	12.75 ± 0.25 B a	16.88 ± 1.46 B b
	7	30.88 ± 2.05 A	28.25 ± 1.39 A	14.25 ± 1.03 B a	18.75 ± 1.24 B b

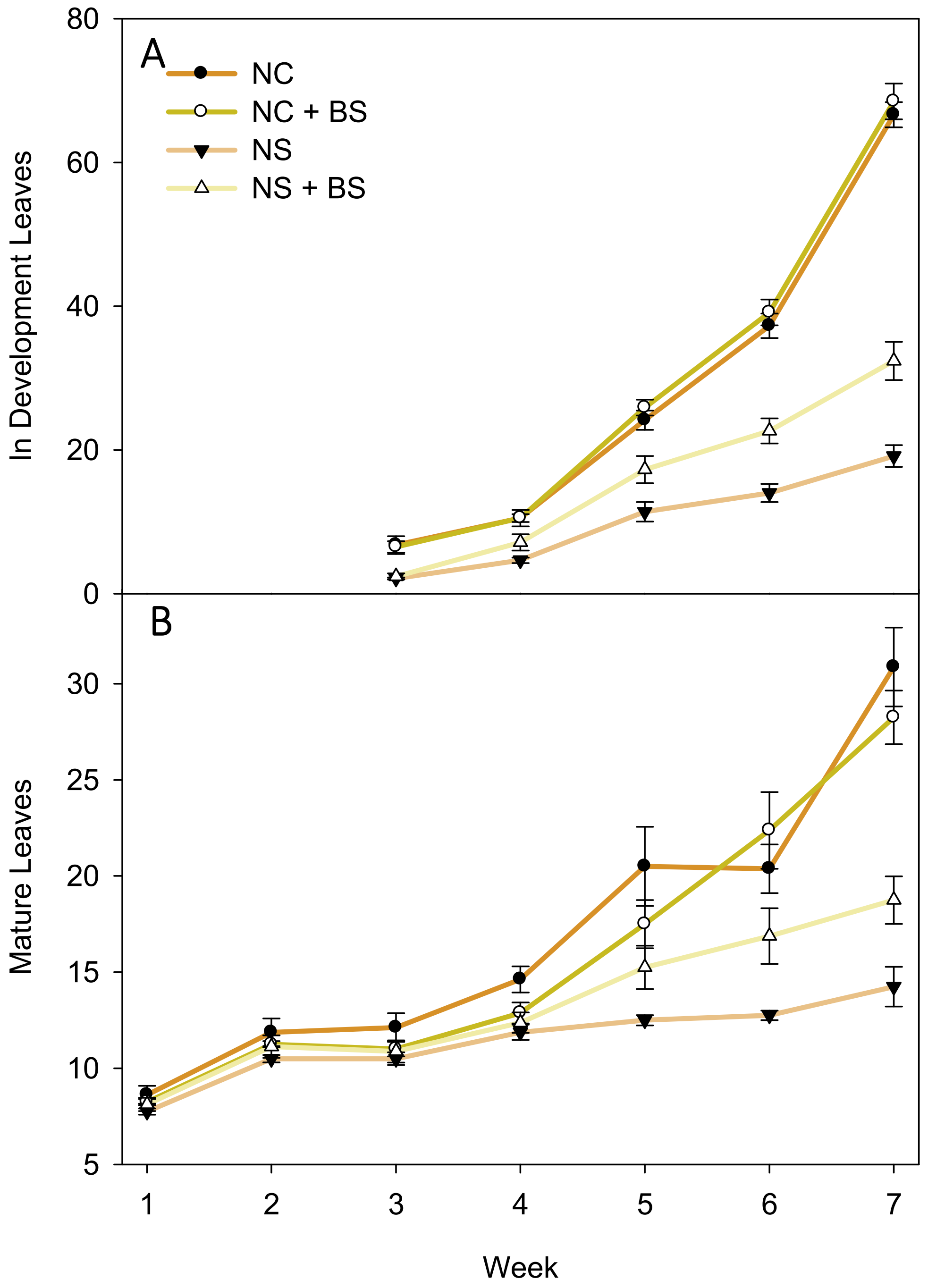

Figure S4. Number of ‘in development leaves’ (A) and ‘mature leaves’ (B) of pepper plants weekly assessed during the plant development first stage (from 1st to 7th week) in the four treatments. NC: Nitrogen Control (circle); NS: Nitrogen Stress (triangle); +BS: Biostimulant applications (empty symbol). Data show the mean ± SE (n=8).

Table S5. Number of flower buds (A) and developed flowers (B) of pepper plants weekly assessed during the plant reproductive stage (from 4th to 11th week) in the four treatments. NC: Nitrogen Control; NS: Nitrogen Stress; +BS: Biostimulant application. Data show the mean ± SE (n=8). Different letters indicate significant differences between nitrogen treatments (capital letters) or between BS applications (lower-case letters) with $P_{value} < 0.05$.

	Week	NC	NC+BS	NS	NS+BS
Flower Buds	4	4.50 ± 1.00 ^{A}	6.38 ± 0.84 ^{A}	2.13 ± 0.44 ^{B}	3.13 ± 0.58 ^{B}
	5	11.63 ± 1.49	12.88 ± 0.69 ^{A}	8.25 ± 1.52	7.88 ± 1.09 ^{B}
	6	19.00 ± 1.44 ^{A}	18.63 ± 0.71 ^{A}	7.88 ± 0.58 $^{B\ a}$	11.13 ± 0.90 $^{B\ b}$
	7	42.63 ± 4.45 ^{A}	42.00 ± 2.19 ^{A}	11.25 ± 0.41 $^{B\ a}$	22.00 ± 0.96 $^{B\ b}$
	8	60.63 ± 2.88 ^{A}	62.75 ± 2.40 ^{A}	11.00 ± 0.82 $^{B\ a}$	25.88 ± 1.01 $^{B\ b}$
	9	75.75 ± 4.17 ^{A}	82.88 ± 2.60 ^{A}	13.13 ± 0.97 $^{B\ a}$	28.63 ± 1.64 $^{B\ b}$
	10	82.88 ± 5.31 ^{A}	90.38 ± 2.28 ^{A}	12.13 ± 2.08 $^{B\ a}$	25.38 ± 1.98 $^{B\ b}$
	11	104.38 ± 4.88 ^{A}	116.00 ± 5.71 ^{A}	10.00 ± 2.09 $^{B\ a}$	21.25 ± 2.30 $^{B\ b}$
Flowers	6	0.75 ± 0.37	0.50 ± 0.27	2.65 ± 0.87	0.25 ± 0.16
	7	3.00 ± 0.63 ^{A}	4.00 ± 0.19 ^{A}	1.00 ± 0.33 ^{B}	1.38 ± 0.46 ^{B}
	8	4.63 ± 0.65 ^{A}	4.75 ± 0.31 ^{A}	1.38 ± 0.42 ^{B}	2.63 ± 0.53 ^{B}
	9	8.25 ± 1.05 ^{A}	9.50 ± 1.02 ^{A}	3.38 ± 0.60 ^{B}	5.50 ± 0.80 ^{B}
	10	14.63 ± 1.13 ^{A}	14.25 ± 0.77 ^{A}	2.25 ± 0.59 $^{B\ a}$	7.00 ± 0.76 $^{B\ b}$
	11	16.63 ± 2.29 ^{A}	19.13 ± 0.69 ^{A}	3.13 ± 0.81 ^{B}	5.88 ± 1.38 ^{B}

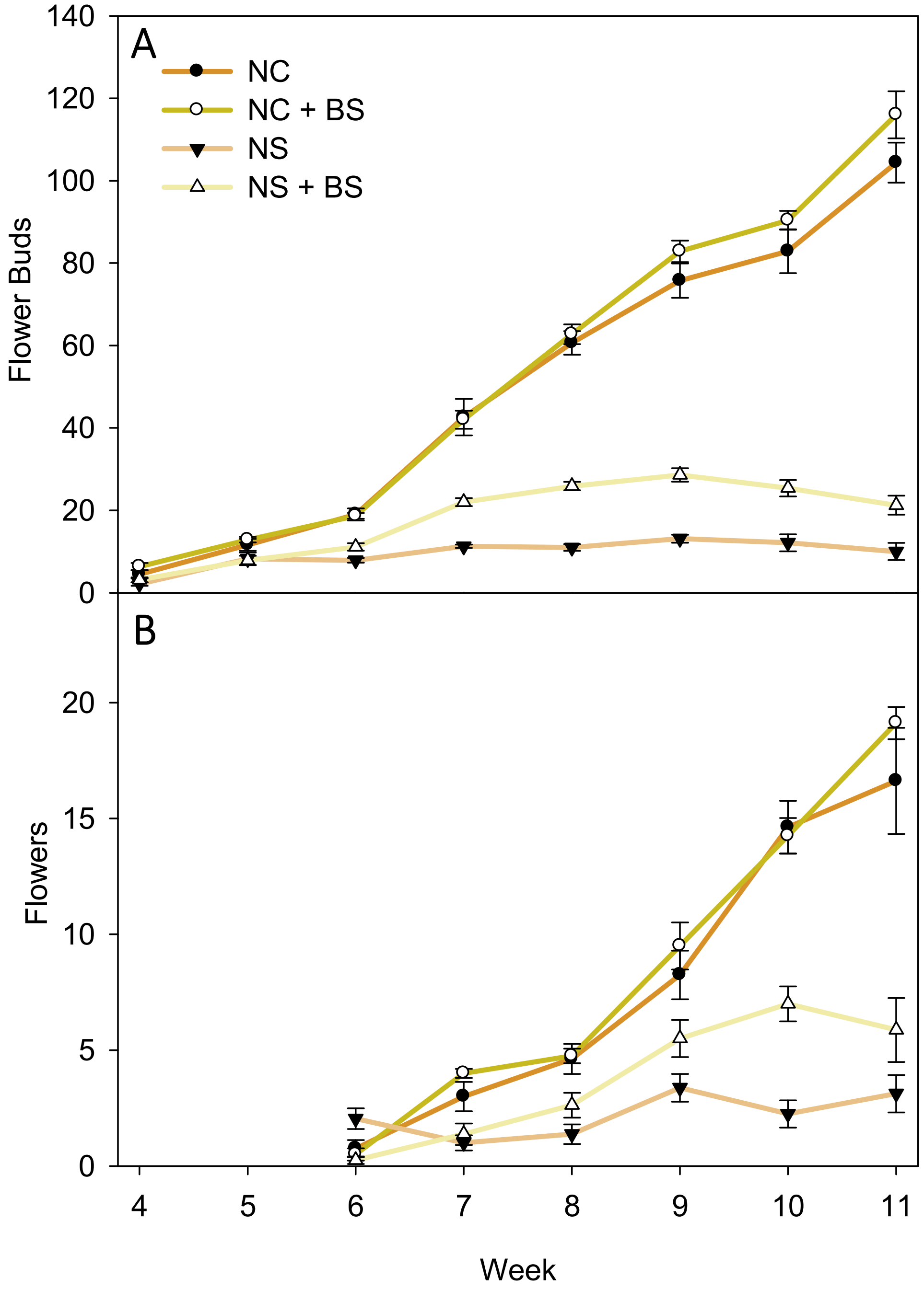

Figure S5. Number of flower buds (A) and developed flowers (B) of pepper plants weekly assessed during the plant reproductive stage (from 4th to 11th week) in the four treatments. NC: Nitrogen Control (circle); NS: Nitrogen Stress (triangle); +BS: Biostimulant applications (empty symbol). Data show the mean ± SE (n=8).

Table S6. Number of just sprouted pepper fruits smaller or equal (A) to 1 cm or bigger (B) than 1 cm weekly assessed during the late plant reproductive stage (from 8th to 11th week) in the four treatments. NC: Nitrogen Control; NS: Nitrogen Stress; +BS: Biostimulant application. Data show the mean ± SE (n=8). Different letters indicate significant differences between nitrogen treatments (capital letters) or between BS applications (lower-case letters) with $P_{value} < 0.05$.

	Week	NC	NC+BS	NS	NS+BS
Just sprouted (≤1cm)	8	2.13 ± 0.52	2.75 ± 0.59	2.00 ± 0.38	2.63 ± 0.60
	9	4.00 ± 0.89 ^{A}	2.50 ± 0.89	1.63 ± 0.38 $^{B\ a}$	3.00 ± 0.42 ^{b}
	10	5.88 ± 0.83 ^{A}	8.00 ± 0.93 ^{A}	1.00 ± 0.27 $^{B\ a}$	5.25 ± 0.65 $^{B\ b}$
	11	12.75 ± 3.03 ^{A}	13.13 ± 1.88 ^{A}	2.00 ± 0.50 $^{B\ a}$	7.00 ± 1.50 $^{B\ b}$
Just sprouted (>1cm)	8	0.75 ± 0.41	0.50 ± 0.27	0.13 ± 0.13	0.13 ± 0.13
	9	1.00 ± 0.50	1.38 ± 0.53 ^{A}	0.25 ± 0.16	0.13 ± 0.13 ^{B}
	10	4.50 ± 1.35 ^{A}	3.50 ± 0.94	1.00 ± 0.33 $^{B\ a}$	2.50 ± 0.53 ^{b}
	11	17.13 ± 1.38 ^{A}	16.50 ± 1.52 ^{A}	0.88 ± 0.30 $^{B\ a}$	4.75 ± 0.92 $^{B\ b}$

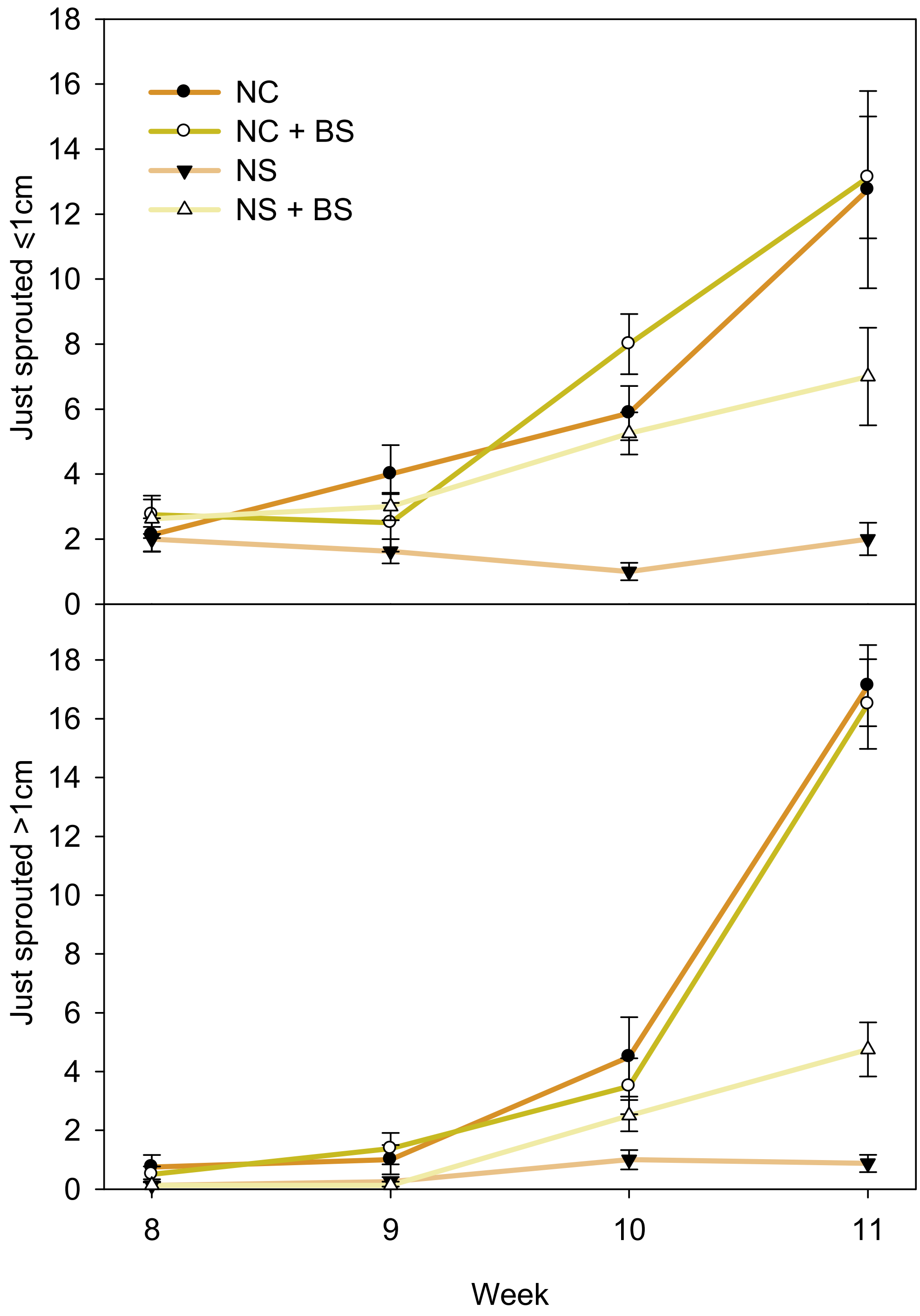

Figure S6. Number of just sprouted pepper fruits smaller or equal (A) to 1 cm or bigger (B) than 1 cm weekly assessed during the late plant reproductive stage (from 8th to 11th week) in the four treatments. NC: Nitrogen Control (circle); NS: Nitrogen Stress (triangle); +BS: Biostimulant application (empty symbol). Data show the mean ± SE (n=8).

Table S7. Chlorophyll content of lettuce plants weekly assessed by SPAD spectroradiometer Index during the whole experiment (from 1st to 5th week) in the four treatments. NC: Nitrogen Control; NS: Nitrogen Stress; +BS: Biostimulant application. Data show the mean ± SE (n=8). Different letters indicate significant differences between nitrogen treatments (capital letters) or between BS applications (lower-case letters) with $P_{value} < 0.05$.

Week	NC	NC+BS	NS	NS+BS
1	26.71 ± 1.17	26.69 ± 0.67	28.21 ± 1.48	26.23 ± 0.59
2	28.45 ± 0.49 a	30.65 ± 0.40 A b	29.53 ± 0.48	28.46 ± 0.54 B
3	30.21 ± 0.64 A	29.48 ± 0.41 A	27.47 ± 0.38 B	28.17 ± 0.38 B
4	33.80 ± 0.81 A	32.45 ± 0.73 A	27.96 ± 0.68 B	29.24 ± 0.41 B
5	37.95 ± 1.12 A	36.97 ± 0.81 A	31.45 ± 0.56 B	32.65 ± 0.32 B

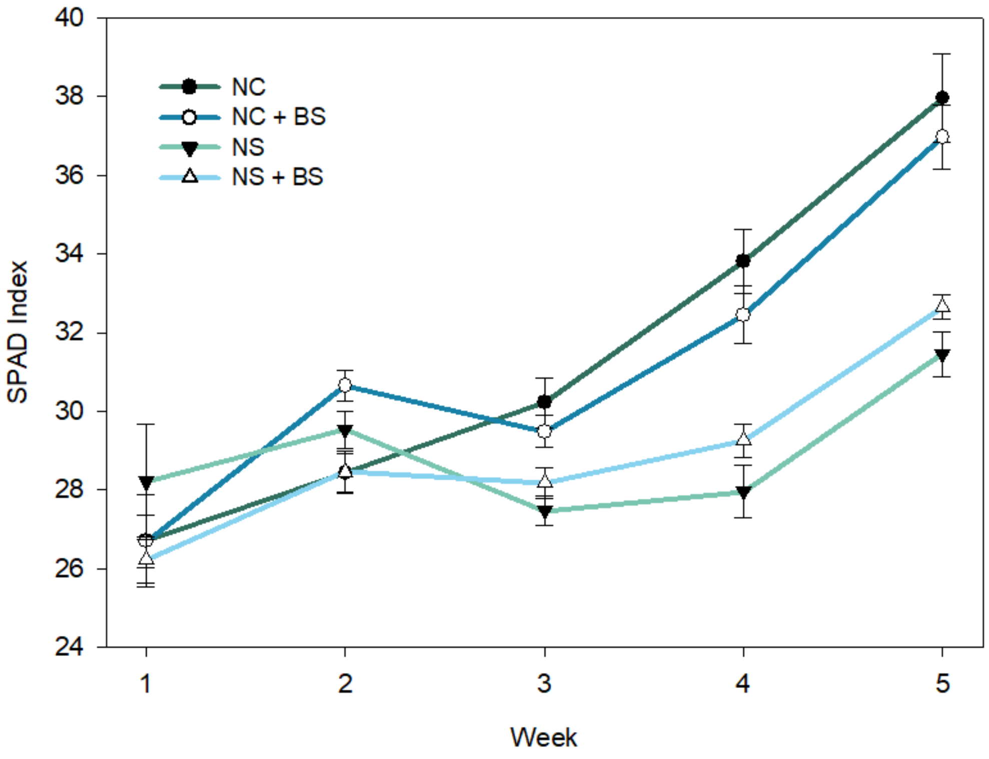

Figure S7. Chlorophyll content of lettuce plants weekly assessed by SPAD spectroradiometer Index during the whole experiment (from 1st to 5th week) in the four treatments. NC: Nitrogen Control (circle); NS: Nitrogen Stress (triangle); +BS: Biostimulant application (empty symbol). Data show the mean ± SE (n=8).

Table S8. Height (A) and number of Mature Leaves (B) of the lettuce plants weekly assessed during the whole experiment (from 1st to 5th week) in the four treatments. NC: Nitrogen Control; NS: Nitrogen Stress; +BS: Biostimulant application. Data show the mean ± SE (n=8). Different letters indicate significant differences between nitrogen treatments (capital letters) or between BS applications (lower-case letters) with $P_{value} < 0.05$.

	Week	NC	NC+BS	NS	NS+BS
Height	1	7.13 ± 0.34	7.18 ± 0.29	7.41 ± 0.19	7.56 ± 0.34
	2	11.21 ± 0.43	11.45 ± 0.27 ^{A}	10.26 ± 0.24	10.15 ± 0.13 ^{B}
	3	12.88 ± 0.27 ^{A}	13.53 ± 0.58	11.68 ± 0.25 ^{B}	12.29 ± 0.42
	4	15.40 ± 0.16 ^{A}	16.31 ± 0.57 ^{A}	13.21 ± 0.42 ^{B}	13.73 ± 0.38 ^{B}
	5	17.10 ± 0.44 ^{A}	17.76 ± 0.46 ^{A}	14.30 ± 0.34 ^{B}	14.90 ± 0.27 ^{B}
Mature leaves	1	5.25 ± 0.16	5.00 ± 0.27	4.88 ± 0.23	4.88 ± 0.23
	2	8.25 ± 0.25	8.25 ± 0.25	8.38 ± 0.38	8.00 ± 0.33
	3	10.13 ± 0.23 ^{A}	9.88 ± 0.23	9.13 ± 0.30 ^{B}	9.13 ± 0.30
	4	14.00 ± 0.38	13.88 ± 0.77	13.13 ± 0.35	13.13 ± 0.35
	5	14.50 ± 0.19	14.88 ± 0.48 ^{A}	12.88 ± 0.95	12.00 ± 0.65 ^{B}

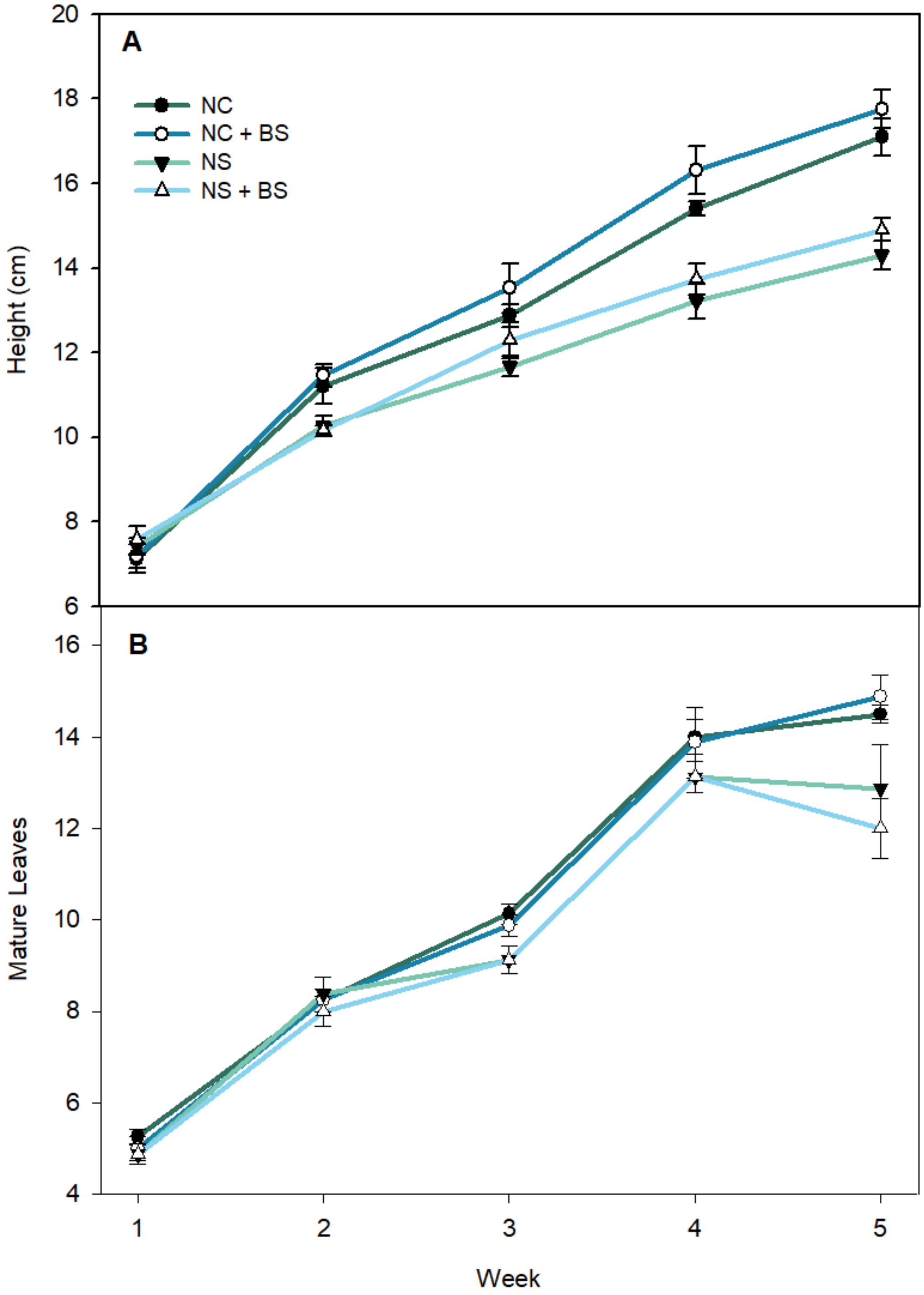

Figure S8. Height (A) and number of Mature Leaves (B) of the lettuce plants weekly assessed during the whole experiment (from 1st to 5th week) in the four treatments. NC: Nitrogen Control (circle); NS: Nitrogen Stress (triangle); +BS: Biostimulant application (empty symbol). Data show the mean ± SE (n=8).
